# The importance of early biomarkers in the identification of neurodegenerative disorders

**DOI:** 10.12688/f1000research.163212.1

**Published:** 2025-07-10

**Authors:** Monica Pinoli, Michele Terzaghi, Franca Marino, Cristoforo Comi, Maurizio Versino, Marco Cosentino

**Affiliations:** 1 Center of Research in Medical Pharmacology, University of Insubria, Varese, Italy; 2 Unit of Sleep Medicine and Epilepsy, IRCCS Mondino Foundation, Pavia, Italy; 3 Movement Disorders Centre, Neurology Unit, Department of Translational Medicine, University of Piemonte Orientale, Novara, Italy; 4 Department of Medicine and Surgery, University of Insubria, Varese, Italy

**Keywords:** Early biomarkers, neurodegenerative disorders, Parkinson’s disease; CD4+ T lymphocytes; Transcription factors; Gene expression

## Abstract

In this datanote we presented the data of 31 iRBD (idiopathic Rapid eye movement (REM) sleep Behaviour Disorder) patients studied throughout three years to assess their eventual phenoconversion to Parkinson’s disease and other established neurodegenerative conditions.

iRBD is a prodromal condition involved in the development of neurological pathologies such as Parkinson’s disease. In a previous study we evaluated transcription factor mRNA levels in CD4+ T cells as predictive biomarkers of phenoconversion in iRBD. We demonstrated that among the transcription factors mRNA levels analysed, STAT1, GATA3 and FOXP3 mRNA levels in CD4+ T cells may be used to predict phenoconversion.

## Introduction

Rapid eye movement (REM) sleep behaviour disorder (RBD) is a REM-phase parasomnia, typically associated with neurodegenerative disorders (38-75% ^1, 2^). iRBD (idiopathic Rapid eye movement (REM) sleep Behaviour Disorder) is a prodromal condition in the process underlying synucleinopathies, including Parkinson’s disease (PD). The risk for PD conversion in people with iRBD is estimated around 43% at five years.

The data included here are from a study ^3^ that evaluated whether transcription factor genes *TBX21*, *STAT1*, *STAT3*, *STAT4*, *STAT6*, *RORC*, *GATA3*, *FOXP3*, and *NR4A2* mRNA levels in CD4+ T cells could represent predictive biomarkers of phenoconversion in iRBD patients. To this end, we performed a follow-up of the iRBD subjects enrolled in previous research, ^4^ with the aim to identify three years later who was eventually phenoconverted towards a frank neurodegenerative condition. The study by Di Francesco et al. (2021) showed that in iRBD subjects CD4+ T cells exhibit a peculiar molecular signature strongly resembling cells from PD patients, while the study by Pinoli et al. (2024) thereafter revealed that STAT1, GATA3 and FOXP3 mRNA levels in CD4+ T cells are promising predictive biomarkers of phenoconversion in iRBD patients.

Data reported in this paper have been previously analysed in Pinoli et al. (2024) and De Francesco et al. (2021).

## Materials and methods

### Ethical statement

The Ethics Committee of the Neurological Institute “C. Mondino” of Pavia approved the protocol (number 2021008499, 10/02/2021) and all the participants signed a written informed consent before enrolment. ^4^

### Participants

31 patients were followed at the Sleep Center of the Neurological Institute “C. Mondino” of Pavia. The clinical evaluation included: motor symptoms, assessed by means of the Unified Parkinson’s Disease Rating Scale (UPDRS) part III ^5^; cognitive function, by the Mini Mental State Examination (MMSE) ^6^; constipation and orthostatic hypotension by means of the Scale for Outcomes in Parkinson’s disease-Autonomic (SCOPA-AUT) ^7^; depressive symptoms, by means of the Hamilton Rating Scale for Depression (HAM-D). ^8^

At the original enrolment, ^4^ each patient provided a venous blood sample, which was processed to isolate CD4+ T cells, according to an established procedure. ^9^

Subjects with iRBD were followed prospectively with periodic in-person evaluations to diagnose phenoconversion, i.e., parkinsonism, DLB and MSA.

### Isolation of CD4+ T Cells

Peripheral blood mononuclear cells were separated by Ficoll-Paque Plus density gradient centrifugation; then CD4+ T cells were obtained by immunomagnetic sorting. Cell viability was 95% ± 1% (range, 90%–98%), and purity was 98% ± 2% (range, 97%–100%). Expression of the TF genes TBX21, STAT1, STAT3, STAT4, STAT6, RORC, GATA3, FOXP3, and NR4A2 was measured by real-time polymerase chain reaction.

### Real-Time Polymerase Chain Reaction Assay

For real-time PCR assays of CD4+ T cells, at least 50,000 CD4+ T cells were resuspended in PerfectPure RNA lysis buffer (5 Prime GmbH, Hamburg, Germany), and total RNA was extracted by PerfectPure RNA Cell Kit™ (5 Prime GmbH, code 2302340). The amount of extracted RNA was estimated by spectrophotometry at λ = 260 nm. Total mRNA was then reverse-transcribed using a random primer and a high-capacity cDNA RT kit (Applied Biosystems, code 4368813), and the resulting amount of cDNA was estimated by spectrophotometry at λ = 260 nm. Real-Time PCR reactions were then started with 1 μM cDNA. At the beginning, we always loaded 1 μg/μl of cDNA per reaction (final reaction mix volume + sample = 20 μl). This means that for each reaction we load 2 μl of aqueous solution containing the cDNA (therefore, 2 μg of cDNA in total). The following thermal protocol was used for each sample: 20 s at 95°C (x 1, hot start); 2-step cycles as follows: 1 s at 95°C, 20 s at 60°C (x 40). All the reagents (probes and mix) were used according to the manufacturer’s instructions ( www.bio-rad.com).

## Ethics and consent

Ethical approval and consent were not required.

## Data Availability

FigShare: “The importance of early biomarkers in the identification of neurodegenerative disorders”. Doi: 10.6084/m9.figshare.28603907 ^10^ The project contains the following underlying data:
• Raw data of patients collected during enrolment and follow-up (.xls) • Patients were labeled with an anonymized sample ID number. Data are available under the terms of the Creative Commons Attribution 4.0 International license (CC-BY 4.0).

## Software availability

GraphPad Prism (version 10.0.0 [computer software] for Windows, GraphPad Software, Boston, Massachusetts USA, www.graphpad.com).
