# Supplementary material for: CD4+ T-cell transcription factors as early predictors of phenoconversion in idiopathic rapid eye movement sleep behaviour disorder
Source: F1000Res. 2025 Oct 6;14:683. Originally published 2025 Jul 10. [Version 2] doi: 10.12688/f1000research.163212.2 (PMC12569504; doi:10.12688/f1000research.163212.2)
Supplement: Peer review history

# Reviewer report: Reviewer response for version 2

**DOI:** 10.5256/f1000research.188922.r420598

**Published:** 2025-10-28

**Authors:** Adawiya J. Haider

**Affiliations:** 1 University of Technology, Baghdad, Baghdad, Iraq

Thanks, authors have made all comments.

 regards

Are sufficient details of methods and materials provided to allow replication by others?

No

Is the rationale for creating the dataset(s) clearly described?

No

Are the datasets clearly presented in a useable and accessible format?

No

Are the protocols appropriate and is the work technically sound?

No

Reviewer Expertise:

Nanotechnology

I confirm that I have read this submission and believe that I have an appropriate level of expertise to confirm that it is of an acceptable scientific standard.

# Reviewer report: Reviewer response for version 2

**DOI:** 10.5256/f1000research.188922.r420597

**Published:** 2025-10-07

**Authors:** Tommaso Schirinzi

**Affiliations:** 1 University of Rome Tor Vergata, Via Cracovia, Roma, Italy

No comments, The manuscript is improved.

Are sufficient details of methods and materials provided to allow replication by others?

Yes

Is the rationale for creating the dataset(s) clearly described?

Partly

Are the datasets clearly presented in a useable and accessible format?

Yes

Are the protocols appropriate and is the work technically sound?

Yes

Reviewer Expertise:

NA

I confirm that I have read this submission and believe that I have an appropriate level of expertise to confirm that it is of an acceptable scientific standard.

# Reviewer report: Reviewer response for version 1

**DOI:** 10.5256/f1000research.179522.r411090

**Published:** 2025-09-17

**Authors:** Adawiya J. Haider

**Affiliations:** 1 University of Technology, Baghdad, Baghdad, Iraq

1. The title needs revision; should the name match the content of the text paper?

 2-Abstract: not clear and should be more scientific and include some of the results in this paper.

 3- The introduction needs expansion in the last paragraph and focuses on the novelty of this work.

 4- This work needs more expansion and agreement with the previous research.

Are sufficient details of methods and materials provided to allow replication by others?

No

Is the rationale for creating the dataset(s) clearly described?

No

Are the datasets clearly presented in a useable and accessible format?

No

Are the protocols appropriate and is the work technically sound?

No

Reviewer Expertise:

Nanotechnology

I confirm that I have read this submission and believe that I have an appropriate level of expertise to confirm that it is of an acceptable scientific standard, however I have significant reservations, as outlined above.

# Response

**Published:** 2025-09-27

**Authors:** Monica Pinoli

**Affiliations:** University of Insubria, Varese, Lombardy, Italy

Dear Dr. Haider,

 Thank you for your valuable suggestions that provided us with insights to improve our manuscript.

 1. The title needs revision → The title has been modified to better describe the content of the paper.

 2-Abstract: not clear and should be more scientific and include some of the results in this paper → The abstract has been extended to include the core results.

 3- The introduction needs expansion in the last paragraph and focuses on the novelty of this work → We changed some parts of the introduction, adding sentences which should better clarify the novelty of this work.

 4- This work needs more expansion and agreement with the previous research → Previous research related to this work has been now more extensively mentioned and discussed to make clearer the novelty and relevance of the present contents.

# Reviewer report: Reviewer response for version 1

**DOI:** 10.5256/f1000research.179522.r399771

**Published:** 2025-08-23

**Authors:** Tommaso Schirinzi

**Affiliations:** 1 University of Rome Tor Vergata, Via Cracovia, Roma, Italy

This manuscript presented a full dataset including clinical and biological data derived from iRBD patients who eventually converted to PD. 

 The effort is valuable and the methodological issues are pretty addressed. 

 I suggest avoiding repeating the RBD abbreviation and indicating references for the statements regarding the risk of phenoconversion. 

 Further, I recommend clearly stating the main aim of this publication.

Are sufficient details of methods and materials provided to allow replication by others?

Yes

Is the rationale for creating the dataset(s) clearly described?

Partly

Are the datasets clearly presented in a useable and accessible format?

Yes

Are the protocols appropriate and is the work technically sound?

Yes

Reviewer Expertise:

Biological issues of PD and movement disorders

I confirm that I have read this submission and believe that I have an appropriate level of expertise to confirm that it is of an acceptable scientific standard.

# Response

**Published:** 2025-09-27

**Authors:** Monica Pinoli

**Affiliations:** University of Insubria, Varese, Lombardy, Italy

Dear Dr. Schirinzi,

 we want to thank you for your suggestions, that help us to improve our manuscript.

1. Avoiding repeating the RBD abbreviation → Repetition of abbreviations have been eliminated.
2. Indicate the reference for the statements regarding the risk of phenoconversion → We added the reference.
3. I recommend clearly stating the main aim of this publication → We modified the introduction and tried to better highlight the aim.
